# Non-Target Screening of Chemicals in Selected Cotton Products by GC/MS and Their Safety Assessment

**DOI:** 10.3390/molecules29153584

**Published:** 2024-07-30

**Authors:** Łukasz Dąbrowski

**Affiliations:** Department of Food Analysis and Environmental Protection, Faculty of Chemical Technology and Engineering, Bydgoszcz University of Science and Technology, 3 Seminaryjna Street, 85-326 Bydgoszcz, Poland; lukas@pbs.edu.pl; Tel.: +48-523749014

**Keywords:** cotton, non-target analysis, textile safety, cotton analysis

## Abstract

Cotton is used for the production of textiles, hygiene and cosmetic materials. During cultivation and technological processes, various types of substances (surfactants, softeners, lubricants, etc.) penetrate cotton, which can have a harmful effect on both the human body and the environment. The aim of this study was to analyze selected cotton products in order to identify the substances contained and to describe the potential possibilities of inducing textile contact dermatitis (CD). The impact of the identified compounds on the aquatic environment was also taken into account. Nine samples of cotton clothing and seven samples of cotton pads from various manufacturers were tested. Samples after extraction using the FUSLE (Focused Ultrasonic Liquid Extraction) technique were analyzed with GC/MS. Qualitative analysis was based on comparing mass spectra with library spectra using the following mass spectra deconvolution programs: MassHunter (Agilent), AMDIS (NIST), and PARADISE (University of Copenhagen). The parameter confirming the identification of the substance was the retention index. Through the non-target screening process, a total of 36 substances were identified, with an average AMDIS match factor of approximately 900 (“excellent match”). Analyzing the properties of the identified compounds, it can be concluded that most of them have potential properties that can cause CD, also due to the relatively high content in samples. This applies primarily to long-chain alkanes (C25–C31), saturated fatty acids, fatty alcohols (e.g., oleyl alcohol), and fatty acid amides (e.g., oleamide). However, there are not many reports describing cases of cotton CD. Information on the identified groups of compounds may be helpful in the case of unexplained sources of sensitization when the skin comes into contact with cotton materials. Some of the identified compounds are also classified as dangerous for aquatic organisms, especially if they can be released during laundering.

## 1. Introduction

Cotton is often used in the textile and cosmetics industries to produce fabrics and hygiene and cosmetic materials. According to the general perception, because cotton yarn is made of natural plant fibers, it is considered a safe and ecological material.

Cotton consists mainly of α-cellulose (88.0–96.5%) [1], as well as small amounts (up to about 1%) of proteins, pectic substances, inorganic substances, organic acids, and other sugars. Additionally, cotton also contains “cotton waxes”, i.e., a group of lipid compounds found on fibers, which include waxes, fats, and resins. The main compounds forming this fraction are long-chain aliphatic alcohols, glycols, glycerols, sterols, α– and β–amyrins, hydrocarbons, fatty acids, esters of fatty acids, and others [1,2,3,4].

In cotton raw material, in addition to natural compounds, there may be many substances introduced artificially during plant cultivation, the technological processes of fiber processing, the preservation of finished products, etc. During operations to which cotton fiber is subjected (such as scouring, spinning, and weaving), lubricants are usually added to improve its mechanical properties [5]. Non-cellulose fractions are removed from fabrics throughout the preparation of the fabric for the bleaching, dyeing, and finishing processes [6], typically using sodium hydroxide [1]. Waxes are saponified in this process, the cotton fiber is softened, and pectin and other compounds are suspended and removed. Other chemical additives used in the processing of fabrics include dyes, biocides, lubricants, fire retardants, and softeners [1,7]. This broad spectrum of compounds (“auxiliaries”) can remain in the final product. For example, as many as 15 groups of compounds have been identified in newly purchased clothing, including phthalates, substituted quinolines, pesticides, surfactants, and others [8]. Similarly, the presence of alkanes, toluene, and aldehydes was found in various cotton clothes [9]. Despite the use of many substances during the production of textiles, information about their presence, as well as the contents of natural substances, usually does not reach the end user [10,11].

For this reason, many studies have been carried out to analyze textiles for harmful chemical compounds. Typical tests include the analysis of selected groups of compounds based on the standards applicable in given countries or following the guidelines provided by organizations granting quality certificates [12,13]. Two approaches are used to analyze contaminants in textiles. Extraction from the sample is carried out using a solution of sodium chloride in water (simulating human sweat) [14,15,16,17,18] or using organic solvents such as dichloromethane, acetonitrile, acetone, hexane, ethyl acetate, toluene, and others [19,20,21,22,23,24]. The most commonly used extraction technique is ultrasound-assisted solvent extraction [10,19,20,22,23,24,25,26,27]. The obtained extract is filtered or centrifuged (sometimes the solvent is exchanged) and then analyzed using gas or liquid chromatography coupled with mass spectrometry [19,23,24,25,27]. The final determination is often preceded by an extract purification step using techniques such as SPE, QuEChERS, etc. [20,21,22,28]. Textile testing methods described in the literature include groups of substances such as polycyclic aromatic hydrocarbons [23], phthalates [24], organophosphate pesticides [14,15,16,17,20], quinolines [22], brominated flame retardants [25], chlorinated paraffins [26], bisphenol A and parabens [27], and others.

In the case of non-target analysis, the extract purification step is usually omitted [10,29]. Various techniques are used for final determinations (for example, DART-MS [30]), although most often these are chromatographic techniques coupled with mass spectrometry. For gas chromatography, a retention index that can be found in databases and the literature is extremely helpful. In the case of liquid chromatography, retention time prediction models based on LogP are most often used [10,31]. In such a situation, however, the difference between the model and real retention time may be up to 2 min [10]. Nowadays, high-resolution mass spectrometry is used for non-target analysis, and the confidence level is determined for the identified substance, e.g., according to Schymanski et al. [10,32]. Obtaining reliable results using low-resolution mass spectrometry requires the use of software that allows the deconvolution of mass spectra and confirmation of the obtained results using another deconvolution computer program [33], comparison of retention indices [34], or reference to information contained in the literature.

Substances present in fabrics can be released by evaporation (if they have a sufficiently high vapor pressure) and washed out during laundering (if they have sufficient solubility in water). Compounds released into the environment pose a potential threat, especially to the aquatic environment [8,35].

Various chemical compounds can be adsorbed through the skin and enter the human body in this way, e.g., many different chemicals from fabrics have been found in urine, blood, and skin [8]. They can also cause textile contact dermatitis (CD) [36,37]. It can be the result of two mechanisms: sensitization (allergic reaction), caused by the body’s immune reactivity to the substance (product), or irritation, caused by the substance or product. Although allergic reactions are relatively rare in textiles made of cotton, they do occur. In a study conducted by de Olando D. G. et al. [38], a case of an immediate allergic reaction on the skin was described when exposed to cotton. In this case, vicilin was selected with limited probability as a substance causing an allergic reaction. Recently, Değer G. et al. [39] described a case of a very severe allergic reaction caused by the presence of cotton (bandage) in an orthopedic splint (Plaster of Paris) placed to stabilize the fracture. However, the cause of this reaction was not given. Other studies have found that benzalkonium chloride, the presence of which was detected in the bandage of the orthopedic splint, is most likely responsible for the development of severe allergic contact dermatitis [40]. Among the substances that cause contact dermatitis (both irritant and allergic), textile fibers, dyes, and chemical finishes are usually mentioned [7,41].

Some of the substances causing textile contact dermatitis are not routinely determined, and only a case study description (such as the examples mentioned above) makes it possible to identify an irritant or allergen present in a textile. What is more, literature reports show that sometimes substances added to cosmetics (i.e., those that are in planned contact with the skin) can cause allergic contact dermatitis [40,42,43]. One way to select substances found in textiles with potentially irritating or allergenic properties is non-target screening analysis. There are relatively few works in the literature on textiles that deal with this type of analysis [9,10,30].

The aim of this work was the qualitative identification of substances present in selected cotton products (clothes, cotton pads) using the GC/MS technique. The potential of the identified substances to cause textile dermatitis and their impact on the aquatic environment were also discussed.

## 2. Results and Discussion

As a result of the analysis of nine samples of cotton clothing (F1–F9 samples) and seven samples of cotton pads (P1–P7 samples) of various manufacturers, a number of chromatograms were obtained. Some of them are presented in Figures S1–S6.

### 2.1. Identification of the Compounds

The identification of compounds in the sample extract was performed according to the algorithm presented in Section 3.4. The decisive parameter for classifying a given compound as “identified” was the consistency of the retention index derived from the chromatogram with the library retention index (NIST17). In the literature, it is generally assumed that the difference between these indices should not be greater than 10 [34]. The retention index database attached to the NIST mass spectra library contains values given as “median +/− deviation”. In some cases, especially when the data are for compounds with high molar masses, “deviation” is not reported because only a single literature value is available. When there are no experimental data in the database, only the estimated retention index is available with a relatively large margin of error, which, in practice, makes it impossible to realistically assess the degree of compliance of the retention index obtained from the chromatogram with the library one. Thus, this parameter, which determines the correct identification of a substance, despite, in the vast majority of cases, fulfilling its function, does not work in the situation described above.

The match of the mass spectrum obtained from the chromatogram with the library mass spectrum was evaluated based on the match factor (MF) parameter: the average MF value from the AMDIS program for the data from Table 1 was 900 (excellent match), and the average score parameter from the MassHunter program (ver. B.08.00) was also high at 85. However, despite the use of a spectral deconvolution algorithm, due to the huge variety of extracted compounds, some of the MFs are slightly lower than those mentioned. In many cases, when it is not possible to obtain results with high MFs and, thus, obtain confirmed results of compound identification, two-dimensional GC [34] or HRMS [30] is used.

The already mentioned compounds with high molar masses are also difficult to identify because their spectra often contain residual peaks (of very low intensity) from the molecular ion. In such cases, the mass spectrum is often interpreted as coming from a compound that is a homolog with a lower molar mass or as a completely different substance (RI may be the decisive factor). This was the case with the analysis of alkanes.

The results of substance identification in the tested samples are presented in Table 1.

### 2.2. Characterization of Identified Compounds

#### 2.2.1. Alkanes

Relatively large amounts of saturated aliphatic hydrocarbons C25–C31, also referred to as paraffin waxes, were found in the samples. Their occurrence can be explained by their natural occurrence in cotton in the waxes fraction (n.b. epicuticular wax of cotton contains hydrocarbons C23–C34) [44]. Alkanes are also found in petroleum oil spray (C15–C50), used as an insecticide in cotton cultivation [45,46,47] and for spraying grain [48]. Moreover, they are a component of lubricating oils used during the mechanical processing of fibers [45].

However, in the tested samples (Table 1), these compounds were mainly found in knitted textile materials (F1–F9 samples), which indicates their probable secondary origin. The estimated maximum peak area for C26–C30 hydrocarbons is in the order of magnitude of 10^7^ for each of the alkanes (Table S1). Their occurrence in fabrics and “100% cotton products” has previously been noted in other papers [8,9]. The migration of these compounds may be evidenced by the fact that alkanes have been identified in human skin surface lipids as derived from direct contamination from the environment [49]. These compounds are characterized by a very high dermal permeability coefficient (which indicates a high ability to penetrate the skin), as well as a very high LogP (and negligible solubility in water and high Koa), which suggests a strong bond with cotton fibers (Table S1).

Alkanes are classified as eye and skin irritants (Table S1), but they do not have skin-sensitizing properties [45]. In the literature, it was found that allergic contact dermatitis is relatively rare concerning petroleum hydrocarbons (C6–C16), for which both model and in vivo studies have been conducted. However, in the case of prolonged or repeated exposure to these compounds, skin irritation may occur, and they may also affect the skin barrier function [45]. Similarly, the CD is rarely observed in the case of skin contact with petrolatum, which consists mainly of saturated aliphatic hydrocarbons [50] and is used as a vehicle for patch testing. There are reports of impurities present in petrolatum, giving rise to sensitization [50], although other studies lack this type of information [51], despite testing petrolatum from different manufacturers [52].

#### 2.2.2. Saturated Fatty Acids

Some of the compounds identified in the tested samples and belonging to the group of carboxylic acids (i.e., nonanoic (pelargonic) acid, decanoic (capric) acid, dodecanoic (lauric) acid, tetradecanoic (myristic) acid, hexadecanoic (palmitic) acid, octadecanoic (stearic) acid) are characterized by varying degrees of skin and eye irritation and toxicity to aquatic life (Table 1 and Table S2). Despite this, they are used in many cosmetics as fragrance ingredients and cleansing and emulsifying agents. In addition, sodium and potassium salts of higher fatty acids are used in soap production. In the textile industry, fatty acids, as their derivatives, are used as emulsifiers and softeners in fiber processing [53].

Acids C9 and C10 have mainly been identified in cotton pads (Table 1) at relatively small peak areas (Table S2). The first of them (nonanoic acid) is classified as a dermatoxin [54] and a corrosive substance that can cause skin irritation [55] and even injury to the skin and permanent eye damage [56]. Acute dermal LD 50 has been observed at concentrations above 5 g/kg (animal experiments) [57]. This naturally occurring compound—in the form of ammonium salt—is used in agriculture as a herbicide. Decanoic acid has been described by the US Environment Protection Agency (EPA) as being of “low concern based on experimental and modeled data” [54]. Nevertheless, in studies conducted on patients with and without acne, this substance was classified as the strongest irritant (next to C8 and C12) among the tested C2–C16 acids [58]. Another report indicates the formation of skin irritation in humans for concentrations of >1% [56]. Acids (C9–C12) are characterized by a dermal permeability coefficient in the range of about 0.04–0.13 cm/h, LogP in the range of 3.4–4.6, and average solubility in water, which suggests the possibility of their release into the aqueous environment.

Peak areas of dodecanoic acid and tetradecanoic acid in the samples were in the order of magnitude of 10^7^ (Table S2). It should be mentioned that they were present only in the F2 sample, in which all the identified fatty acids were found. C16 and C18 fatty acids are used in the textile industry for fabric softening [59]. Saturated fatty acids have wax-like properties, and in some cases, stearic, palmitic, and myristic acids can function as waxes to achieve water repellency [60]. These compounds are present in all tested samples, and their peak areas are at least an order of magnitude greater compared to other acids. Both acids are classified as mild irritants or even non-irritants [54,56] (C18 acid occurs naturally in human fats).

#### 2.2.3. Fatty Alcohols

Long-chain alcohols occur naturally in cotton wax [3]. Due to their “waxy nature” [60], they can act as non-ionic surfactants [61] when used as additives during fiber processing. Many of the long-chain alcohols are used in the textile industry as surfactants, softeners (to increase viscosity), and lubricants [54,56,62,63].

In the tested samples, C12 and C14 alcohols had relatively small peak areas (Table S3), C16 and C18 alcohols were an order of magnitude larger, and octacosanol had two orders of magnitude larger peak areas, which can be explained by its natural occurrence in cotton wax [64].

Concerning the effects on the human skin, it is assumed that C6–C11 alcohols cause irritation, C12–C16 alcohols are moderately irritating, and C18 and above are non-irritating [65]. In the production of cosmetics, fatty alcohols act as surfactants and detergents to increase their viscosity and foaming capacity and as emulsifiers.

The presence of (9Z)-octadecene-1-ol (oleyl alcohol) with a relatively high maximum peak area with an order of magnitude of 10^8^, a compound referred to as skin sensitizer [54], was detected in cosmetic pads. Most of the identified compounds are also irritating to the eye and toxic to aquatic organisms [54], although their solubility in water is relatively low (Table S2).

#### 2.2.4. Fatty Acid Esters

Fatty acid esters can be used in the textile industry as assistant materials, in particular as softeners [66]. Most of the identified compounds did not have strong adverse effects on the skin (Table S4). The exception is the hexanedioic acid bis(2-ethylhexyl) ester (only in the W5 sample), which is a skin and eye irritant and can also have effects on the central nervous system and liver [54,56]. Lanolin (Table S4) is a substance found in most of the tested samples. This substance is commonly used in cosmetics to moisturize the skin, hair, and nails. Nevertheless, it has been found that it can cause allergic contact dermatitis [67]. Stearyl stearate has been identified in several products (Table S4).

In studies conducted on the development of microorganisms on the skin, depending on the cosmetics used, it was found that fatty acids and fatty esters (from alcohols C12 and above) had an influence on the growth of the tested bacteria. This information is especially important for people with dermal diseases like acne vulgaris [68]. Referring to cotton products, it can be expected that the environment existing in fabrics (containing the aforementioned groups of compounds) will potentially be conducive to the development of microorganisms. Antimicrobial agents added to fabrics protect them for some time, but the activity of these agents decreases after each wash [69].

#### 2.2.5. Phthalate Esters

Phthalates are found in many samples—they are commonly found in the environment and many industrial and personal products (e.g., dibutyl phthalate has been found in personal care and healthcare products [30]). Although phthalates can be biosynthesized by plants [70], their main source is industrial production. They are used as plasticizers and fillers for plastics, as well as viscosity adjusters. In most samples, only two substances belonging to this group were identified: diisobutyl phthalate ester (DIBP) and dibutyl phthalate (DBP). They can be absorbed by the skin and have skin- and eye-irritating properties. In addition, they are endocrine disruptors. They have a negative impact on aquatic organisms (Table S5). Their peak areas in the tested samples are in the order of magnitude of 10^7^. The literature contains information about the relatively high contents of phthalates in textiles, e.g., the contents of phthalate esters in baby waterproof fabrics, decorated waterproof tarpaulins, and printed textiles exceed 0.1 percent [71,72]. Phthalates are a relatively well-studied group of compounds in terms of their effects on human health [73]. There are numerous literature reports indicating that contact dermatitis is a result of exposure to DBP contained in ointments, plastic watchstraps [74], and household dust, as well as exposure of the child to phthalates during fetal life [75]. In the literature (review and research papers), numerous examples of allergic diseases caused by phthalates have been cited [8,73,75,76].

Exposure to phthalates can also occur through airways and the skin [75], but in the case of DIBP and DBP, their low volatility and high Koa value (Table S5) do not indicate a major contribution of this pathway to the skin uptake of these compounds.

#### 2.2.6. Fatty Acid Amides

Hexadecanamide (palmitamide), octadecanamide (stearamide), and 9Z-octadecenamide (oleamide) are fatty amides (metabolites) derived from the corresponding fatty acid. These are natural products found in many plants. Oleamide is used in industry as a lubricating oil and slipping agent in plastic [54,77]. Of the samples tested, it was present only in the F1 sample. This compound is classified as a skin and strong eye irritant, which may cause allergic skin reactions (Table S6). There are reports in the literature about a strong urticarial reaction after exposure to oleamide [78], as well as about the relatively easy migration of this compound from plastics to the environment [79]. It is classified as hazardous to the aquatic environment, which has long-lasting harmful effects on aquatic life (Table 1), although its solubility in water (like other fatty acid amides) is low (about 0.05 mg/L)—Table S6. This compound is found in a large number of household cleaning products, where it acts as a surfactant and is referred to as a “substance of caution” [77], and in personal care and healthcare products [30]. Palmitamide has similar properties; however, is not described as a “strong irritant”. Octadecanamide’s effects on the skin and the environment are somewhat similar to those of palmitamide, although there are no reports of its harmfulness to aquatic organisms [54].

#### 2.2.7. Low-Molecular-Mass Compounds

Taking into account the types of samples being determined, there is a high probability of the evaporation of compounds with low molar masses during the storage of finished products and their display on store shelves. Moreover, the sample preparation procedure consisting of evaporating the extract to dryness could also cause a partial loss of these compounds, despite the use of a method without temperature compensation (Section 3.2).

In a few samples, compounds with low molar masses (<150 amu) were identified, These substances were benzyl alcohol, 2-propylheptane-1-ol, 2-phenoxyethanol, and benzothiazole occurring at low peak areas in the tested samples (Table S7). These compounds can act as preservative agents and are characterized by high solubility in water (up to 2.67 × 10^4^ mg/L), and thus also in human sweat when wearing clothes. They have irritating properties, and some of them are allergens (Table S7). The dermal permeability coefficient for these compounds is low.

#### 2.2.8. Other Compounds

In the analyzed samples, compounds not belonging to the previously described groups were also identified. One of them is dimanthine, which causes severe skin burns and eye damage and is very toxic to aquatic life. It is an ingredient of lubricants and greases [80]. It was identified in sample F3, and the peak area was in the order of magnitude of 10^7^ (Table S8). Another compound identified was tributyl acetyl citrate, which is the most common compound found in household cleaning products [77]—it is used in industry as a biodegradable plasticizer [80]. It has also been found in cotton pads and panty liners [81]. However, it is not classified as a harmful agent [54,80,82]. Similarly, Lureth-4 [54], identified in two samples (Table 1), is found to be safe for use in cosmetics. A commonly identified compound in samples was γ-sitosterol (Table S8). This was—apart from sample F1—the compound with the largest peak areas in the chromatograms. This substance is used in the treatment of diabetes, but no information on the effect of the gamma isomer on human skin has been found in the literature. The beta-sitosterol isomer is used in the production of cosmetics and pharmaceuticals. Sitostenone (with slightly lower peak areas compared to γ-sitosterol), present in the F2 sample, is a natural substance, belongs to the group of sterols, and is used in the pharmaceutical industry [29]. This compound has also been identified in samples of personal care and healthcare products [30].

**Figure 1:**
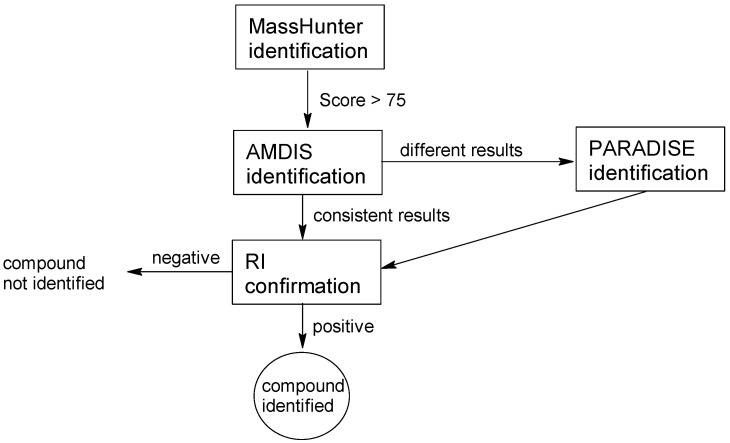
The compound identification algorithm.

During the non-target analysis, many tentatively identified and unidentified compounds were also detected (based on the algorithm in Figure 1), which require further investigation. Some of them show significant abundances (high peak areas), which is an indication of possible higher concentrations. However, their identification would require the use of complementary methods (e.g., LCMS, multidimensional chromatography) to confirm the tentative results. The detected chemicals are not permanently bound to cotton fibers and, therefore, have the potential to migrate to human skin or the environment.

## 3. Materials and Methods

### 3.1. Materials and Reagents

Chromatographic-grade dichloromethane was purchased from Honeywell Riedel-de Haën (Seelze, Germany). The mixture of C7–C40 saturated hydrocarbon standards was purchased from Merck (Poznań, Poland). Samples of undyed (white) fabrics were taken for analysis, which—according to the manufacturer’s declaration—were made of 100% cotton, except sample F3. The samples were purchased in various stores in northern Poland. More detailed information on the samples is given in Table 2.

### 3.2. Sample Preparation

The sample preparation procedure was analogous to the one previously described in the literature [10,22], with minor changes. Samples of the 1 g material were cut into pieces measuring about 0.5 × 0.5 cm. Each sample was placed in a 40 mL glass vial. Then, 12 mL of dichloromethane was added and extracted for 10 min (cycle 0.5, amplitude 100%) using the FUSLE (Focused Ultrasonic Liquid Extraction) technique with the Ultrasonic Processor UP100H (Hielscher Ultrasonics, Teltow, Germany). The second stage of extraction (with 4 mL of dichloromethane) was carried out under the same conditions. The extracts, after filtration through a pipette filter (Mettler-Toledo, Warsaw, Poland), were combined and then evaporated in a gentle stream of nitrogen, without temperature control of the vial, following the previous results obtained for the solvent evaporation process [83]. The dry residue was dissolved in 300 μL DCM and subjected to GC/MS analysis. Sixteen samples together with procedural blanks (whole-procedure tests, solvent purity tests, ultrasonic probe purity tests) were prepared and analyzed in duplicates.

### 3.3. GC/MS Analysis

GC/MS analysis was performed using the 7890B gas chromatograph and the 5977B mass spectrometer (Agilent, Santa Clara, CA, USA). A ZB-5MS chromatographic column with dimensions of 30 m × 0.25 mm × 0.25 μm was used. Helium was used as a carrier gas, with a flow rate of 1.5 mL/min. Samples with volumes of 3 μL were injected into the chromatographic column in the Pressure Pulsed Splitless Injection mode (initial pressure 0.2 MPa (30 p.s.i.) for 1.3 min, decreased to constant flow). Samples were additionally injected in volumes of 1 µL and 0.5 µL (splitless) to confirm the results when identifying compounds with high concentrations in the sample (due to GC/MS overload). The injector was operated at a temperature of 290 °C. Chromatographic analysis was carried out using the oven temperature program: 50 °C (1.5 min), increment of 10 °C/min to 150 °C, and then increment of 5 °C/min to the final temperature of 310 °C (19 min). The mass spectrometer was operated in SCAN mode in the range of 45–650 amu.

### 3.4. Data Processing and Compound Identification Algorithm

The obtained chromatograms were analyzed using the Mass Hunter (MH) Qualitative Workflows ver. B.08.00 (Agilent, Santa Clara, CA, USA), using a compound identification algorithm based on mass spectra deconvolution. If a substance with a SCORE spectrum match (concerning the library spectrum) of at least 75 was identified, the result was confirmed using AMDIS software (version 2.73, NIST, Gaithersburg, MD, USA).

When the identification results obtained from the MH program were not consistent with those from the AMDIS program, the chromatogram was additionally analyzed using PARADISE software, ver.6.0.1 (Figure 1). In all cases, the NIST 17 mass spectra library was used. The parameter determining the recognition of the library search as “correct” was the consistency of the retention index determined from the chromatogram with the library retention index (NIST). Examples of the identification algorithm applied to identified compounds are provided in the Supplementary Materials. The C7–C40 hydrocarbon mixture was used to determine the retention indices of the peaks on the chromatogram (Figure S7). The highest abundance of substances in the analyzed samples was presented as a colored rectangle, which corresponds to the order of magnitude of the peak areas (indicating the possible concentration) of chemical substances (Tables S1–S8).

## 4. Conclusions

As a result of GC/MS analysis of selected cotton products (cotton pads, textiles), 36 compounds were successfully identified. The use of an algorithm using mass spectra deconvolution software (MassHunter, AMDIS, PARADISE) and retention indices enables the analysis of a wide range of compounds present in the extract: from low masses to those over 400 amu. The tested samples are characterized by certain similarities in terms of composition: almost all samples were found to contain alcohols (C12, C14, C16, C28), fatty acids (C9, C10, C16, C18), and phthalates (DBP, DIBP). In the case of other substances, they were present only in some, sometimes single samples, most often in samples of cotton fabrics (e.g., F1, F2), which may indicate their secondary origin (i.e., they are added in the production of the fabrics). To the best of the authors’ knowledge, there are no non-target studies in the literature on samples of undyed cotton materials in terms of the potential negative effects of the substances that they contain. From this perspective, the research presented in this work constitutes scientific novelty.

A large number of the compounds identified in cotton are characterized by a negative impact on aquatic organisms (saturated fatty acids, fatty alcohols, phthalate esters, fatty acid amides). Despite their usually low solubility in water, they can potentially enter the wastewater during subsequent washings. Compounds identified in the samples, both of natural origin and added in the production cycle, are often bioactive compounds. Analyzing their properties, it can be concluded that most of them have potential properties that may cause textile contact dermatitis, also due to their relatively high contents in samples. This applies primarily to long-chain alkanes, saturated fatty acids, fatty alcohols, and fatty acid amides. Although there are not many reports of cases of cotton CD, in the case of unexplained causes of allergy due to contact with cotton clothing or cotton pads, further dermatological studies could take into account the groups of compounds identified in this study.

## Figures and Tables

**Table 1:** A list of the substances identified in the samples: retention indices—RI from chromatogram (chr) and database (DB); score parameter from MassHunter—MH; match factor from PARADISE software (PARADISE) and AMDIS; samples in which the substance was found—samples.

No	RT [min]	Name	CAS RN	RI Chr	RI DB	MH (PARADISE)	AMDIS	Samples
1	7.05	benzyl alcohol	100-51-6	1035	1036 ± 4	93	950	F:5
2	9.80	2-propylheptan-1-ol	10042-59-8	1212	1294 ^ev^	(914)	940	F:5,7
3	9.92	2-phenoxyethanol	122-99-6	1222	1225 ± 3	90	916	F:2,5
4	10.10	benzothiazole	95-16-9	1234	1229 ± 8	91	958	F:2
5	10.66	nonanoic acid	112-05-0	1272	1273 ± 7	81	923	F:2,8; P:1,2,5–7
6	11.96	n-decanoic acid	334-48-5	1364	1373 ± 6	80	923	F^t^:2,8; P^t^:1,2,5–7
7	13.59	dodecan-1-ol	112-53-8	1474	1473 ± 4	88	951	all, except: F:3,4
8	15.11	dodecanoic acid	143-07-7	1568	1568 ± 3	79	854	F:2
9	17.03	tetradecan-1-ol	112-72-1	1677	1676 ± 4	89	940	F:6,9; P:1,2,5–7
10	18.72	tetradecanoic acid	544-63-8	1770	1768 ± 5	86	915	F:2
11	20.36	diisobutyl phthalate	84-69-5	1859	1870 ± 4	94	939	F:1–3; P:1,2,5–7
12	20.83	hexadecan-1-ol	36653-8-4	1882	1880 ± 3	86	958	F:2,5–7; P:1–3,5–7
13	22.11	dibuthyl phthalate	84-74-2	1954	1965 ± 6	92	962	F:1–3,8; P:1,2,4–6
14	22.37	hexadecanoic acid	57-10-3	1969	1968 ± 7	82	958	F:all; P:all
15	24.14	9-octadecen-1-ol, (Z)-	143-28-2	2064	2063 ± 3	90	955	P:1,3,5–7
16	24.99	dimantine	124-28-7	2115	2096 ± na	83	921	F:3
17	24.53	octadecane-1-ol	112-92-5	2086	2082 ± 2	91	964	F:1,2,5,6,9; P:1,3,7
18	25.92	octadecanoic acid	57-11-4	2168	2172 ± 7	85	945	F:all; P:all
19	26.09	hexadecanamide	629-54-9	2179	2184 ± 2	83	873	F:1,6
20	29.28	9-octadecenamide, (Z-)	301-02-0	2363	2386 ± 11	80	892	F:1
21	29.66	octadecanamide	124-26-5	2392	2374 ± 25	76	834	F:3–6
22	29.59	hexanedioic acid bis(2-ethylhexyl)ester	103-23-1	2387	2398 ± 16	89	922	P:5
23	31.40	pentacosane	629-99-2	2502	2500	80	900	F:1,9
24	31.76	hexadecanoic acid 2-hydroxy-1-(hydroxymethyl)ethyl ester	23470-00-0	2526	2519	89	685	F:8
25	32.93	hexacosane	630-01-3	2603	2600	77	928	F:1–9; P:7
26	34.39	heptacosane	593-49-7	2703	2700	(886)	882	F:1–6,8,9; P:7
27	35.80	octacosane	630-02-4	2803	2800	(911)	891	F:1–6,8,9
28	37.16	nonacosane	630-03-5	2902	2900	(786)	850	F:1–9; P:1–3,5,7
29	38.48	triacontane	638-68-6	3008	3000	(706)	890	F:1–9; P^t^:1–7
30	39.72	hentriacontane	630-04-6	3100	3100	(840)	744	F:1,9
31	40.14	octacosan-1-ol	557-61-9	3132	3118 ± 2	87	949	F:2–8; P:all
32	42.48	γ-sitosterol	83-47-6	3312	3221 ± 31	64 (930)	955	F:2–8; P:all
33	42.96	hexadecanoic acid hexadecyl ester	540-10-3	3367	3364 ± na	89	936	F:6,7; P:3,4
34	43.76	sigmast-4-en-3-one	1058-61-3	3434	3447 ± 12	86	862	F:2
35	45.50	hexadecanoic acid, octadecyl ester	2598-99-4	3568	3546 ± na	77	866	F:1,2,4,9; P:2–5,7
36	48.83	octadecanoic acid octadecyl ester	2778-96-3	3767	3764 ± na	83	873	F:2,9; P:2,5

^t^—traces; ^ev^—evaluated value; na—not available.

**Table 2:** Types and origins of the analyzed samples.

Sample Label	Sample Type	Country	Notes
F1	cotton towel	China	Disposable face washing towel
F2	panties	Bangladesh	
F3	children’s panties	China	Bio cotton, 5% elastan
F4	boys’ t-shirt	Bangladesh	
F5	children’s panties	China	“wash before use” on the label
F6	children’s panties	Bangladesh	Organic cotton
F7	cotton sticks	China	
F8	men’s T-shirt	Bangladesh	
F9	men’s shirt	Slovakia	
P1	cotton pads	EU	
P2	cotton pads	EU	
P3	cotton pads	France	Bio cotton
P4	make-up removal wipes	China	
P5	cotton pads	EU	
P6	cotton pads	Poland	
P7	cotton pads	Poland	Organic cotton

## Data Availability

The original contributions presented in the study are included in the article/Supplementary Material, further inquiries can be directed to the corresponding author.

## Supplementary Materials

The following are available online at https://www.mdpi.com/article/10.3390/molecules29153584/s1, Figure S1. GC/MS chromatograms of the sample extract (from the top): F1, F2, F3; Figure S2. GC/MS chromatograms of the sample extract (from the top): F4, F5, and F6; Figure S3. GC/MS chromatograms of the sample extract (from the top): F7, F8, and F9; Figure S4. GC/MS chromatograms of the sample extract (from the top): P1, P2, and P3; Figure S5. GC/MS chromatograms of the sample extract (from the top): P4, P5, and P6; Figure S6. GC/MS chromatogram of the P7 sample extract; Figure S7. GC/MS chromatogram of the n-alkane mixture sample extract; C = 10 µg/mL. The chromatogram shows peaks for C9–C40 (the first two alkanes are missing due to solvent delay); A. Examples of the identification algorithm applied to an identified compound; Table S1. Properties of the identified compounds (alkanes) and the sample with the highest abundance (peak area). The colored rectangle (simplified heat map—SHM) corresponds to the order of magnitude of the peak area; Table S2. Properties of the identified compounds (saturated fatty acids) and the sample with the highest abundance (peak area). The colored rectangle (simplified heat map—SHM) corresponds to the order of magnitude of the peak area; Table S3. Properties of the identified compounds (fatty alcohols) and the sample with the highest abundance (peak area). The colored rectangle (simplified heat map—SHM) corresponds to the order of magnitude of the peak area; Table S4. Properties of the identified compounds (fatty acid esters) and the sample with the highest abundance (peak area). The colored rectangle (simplified heat map—SHM) corresponds to the order of magnitude of the peak area; Table S5. Properties of the identified compounds (phthalate esters) and the sample with the highest abundance (peak area). The colored rectangle (simplified heat map—SHM) corresponds to the order of magnitude of the peak area; Table S6. Properties of the identified compounds (fatty acid amides) and the sample with the highest abundance (peak area). The colored rectangle (simplified heat map—SHM) corresponds to the order of magnitude of the peak area; Table S7. Properties of the identified compounds (low-molecular-mass compounds) and the sample with the highest abundance (peak area). The colored rectangle (simplified heat map—SHM) corresponds to the order of magnitude of the peak area; Table S8. Physicochemical properties of the identified compounds (other compounds) and the sample with the highest abundance (peak area). The colored rectangle (simplified heat map—SHM) corresponds to the order of magnitude of the peak area.
