# Supplementary material for: Non-Target Screening of Chemicals in Selected Cotton Products by GC/MS and Their Safety Assessment
Source: Molecules. 2024 Jul 30;29(15):3584. doi: 10.3390/molecules29153584 (PMC11314172; doi:10.3390/molecules29153584)
Supplement: Supplementary file 1 [file molecules-29-03584-s001.zip › molecules-3008017-supplementary.pdf]

Supplementary materials

Non-target screening of chemicals in selected cotton products by GC/MS and their safety assessment

Łukasz Dąbrowski

Department of Food Analysis and Environmental Protection, Faculty of Chemical Technology and Engineering, Bydgoszcz University of Science and Technology, 3 Seminaryjna Street, 85-326 Bydgoszcz, POLAND; lukas@pbs.edu.pl; Tel.: +48 523749014

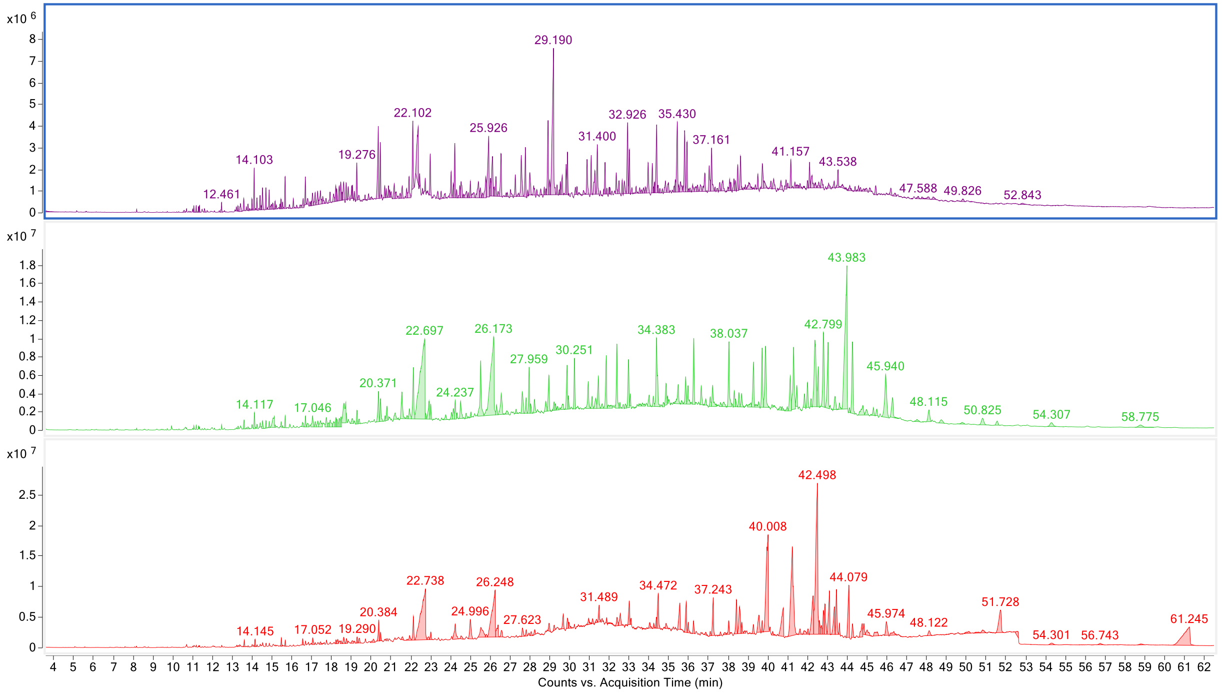

Figure S1. GC/MS chromatograms of the sample extract (from the top): F1, F2, F3

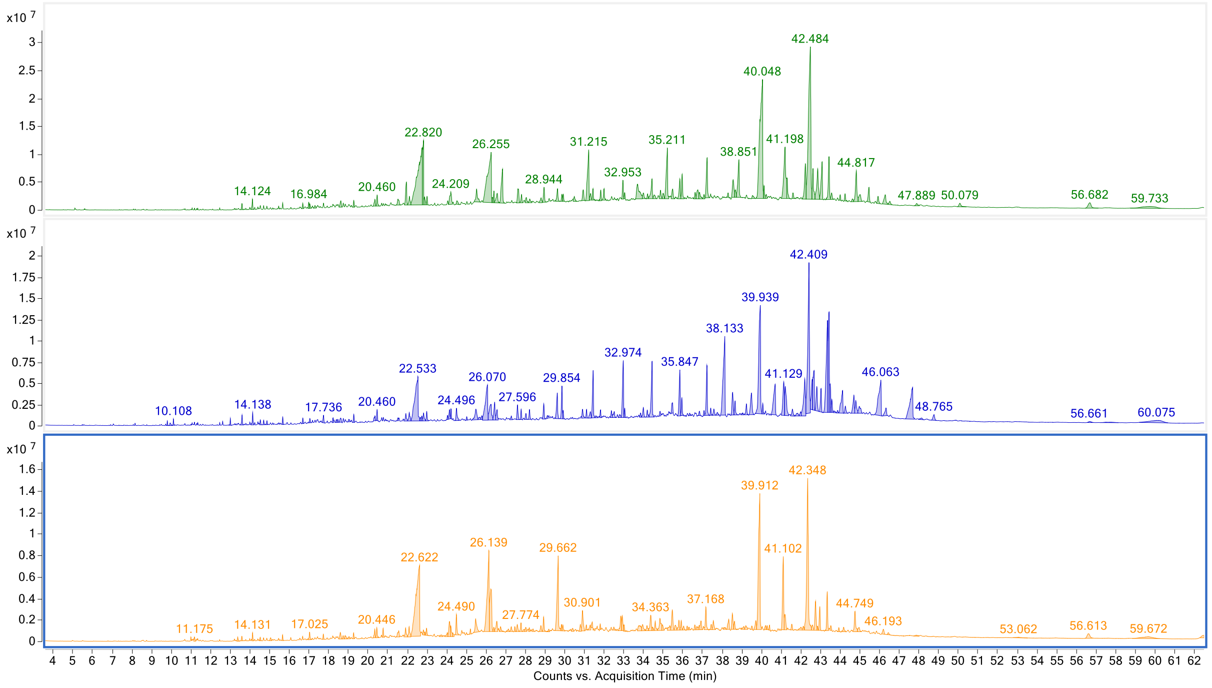

Figure S2. GC/MS chromatograms of the sample extract (from the top): F4, F5, F6

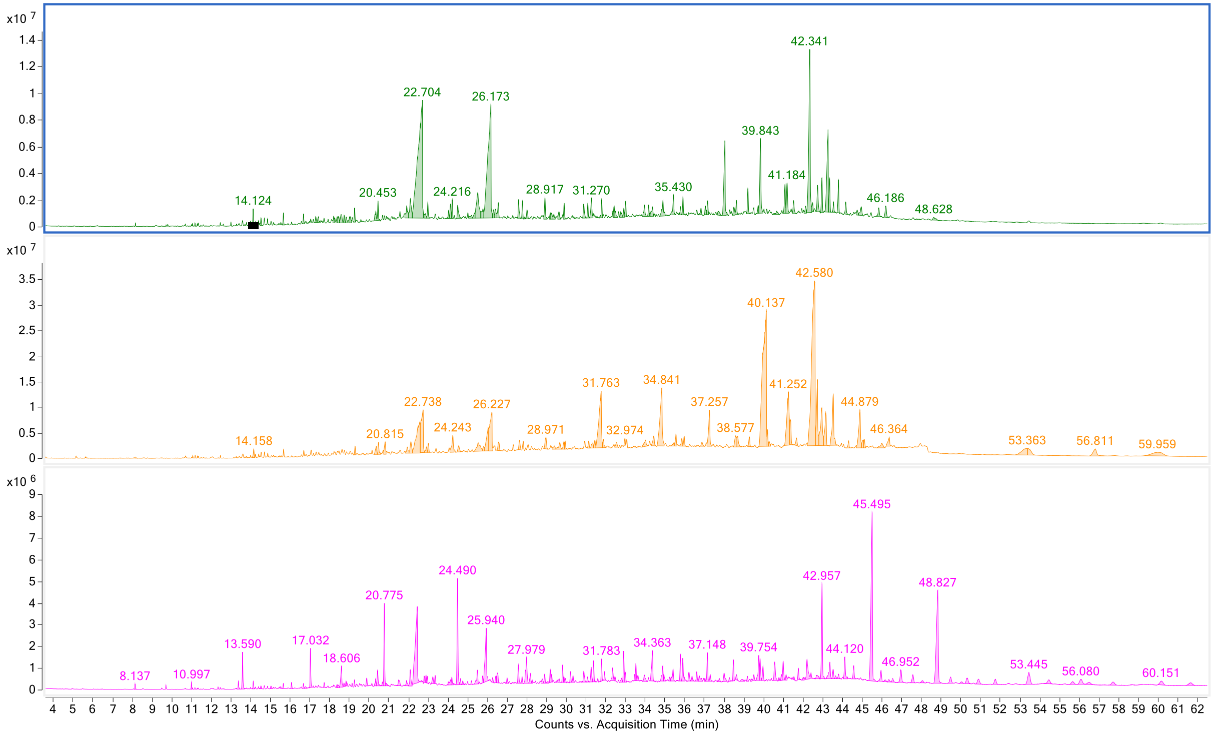

Figure S3. GC/MS chromatograms of the sample extract (from the top): F7, F8, F9

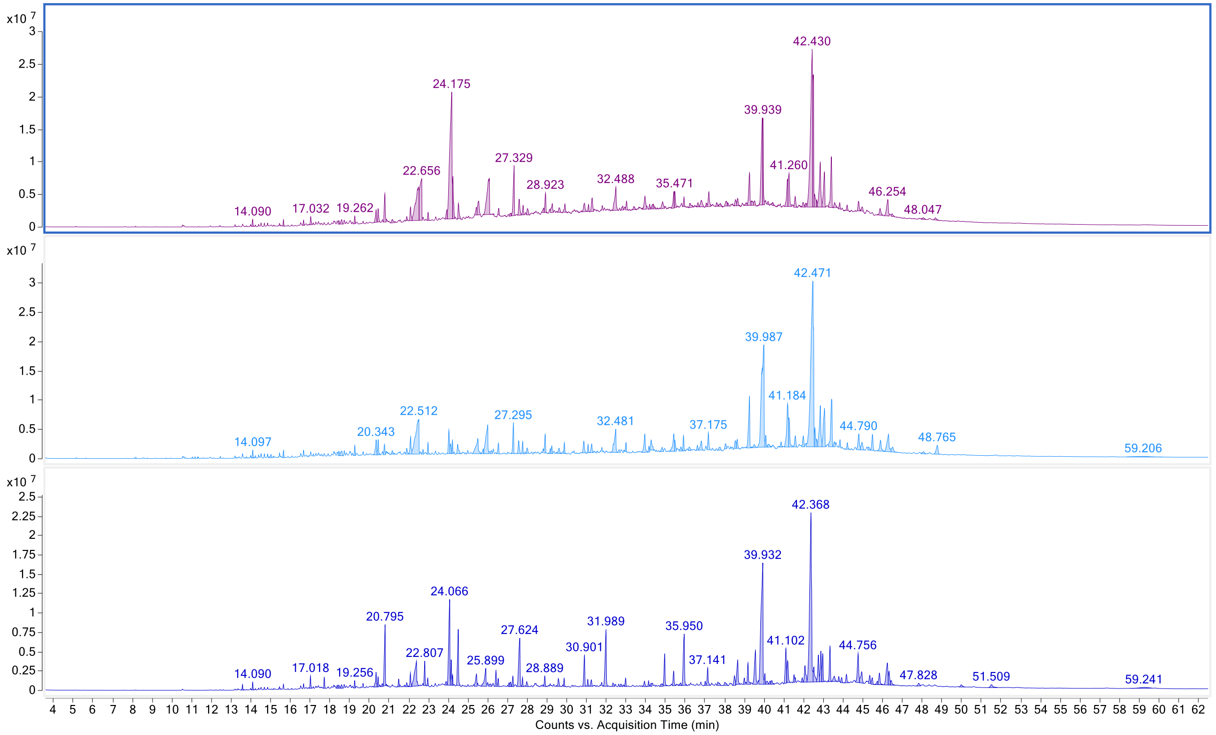

Figure S4. GC/MS chromatograms of the sample extract (from the top): P1, P2, P3

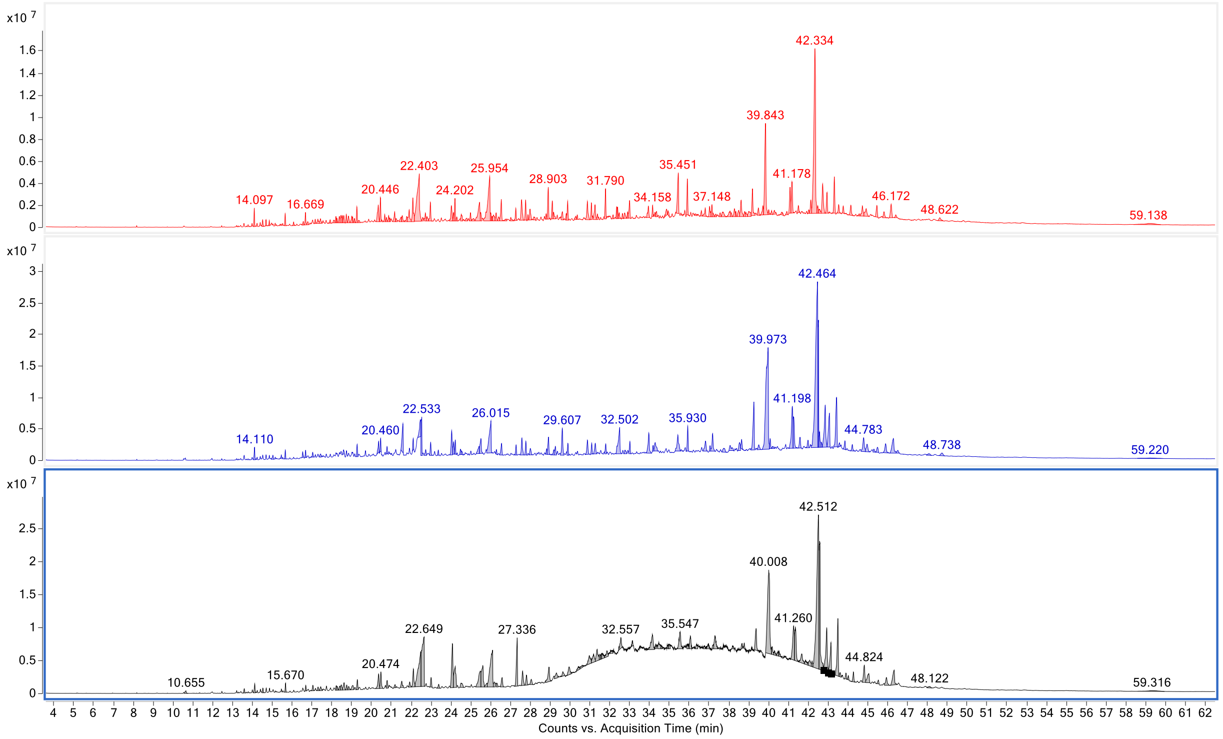

Figure S5. GC/MS chromatograms of the sample extract (from the top): P4, P5, P6

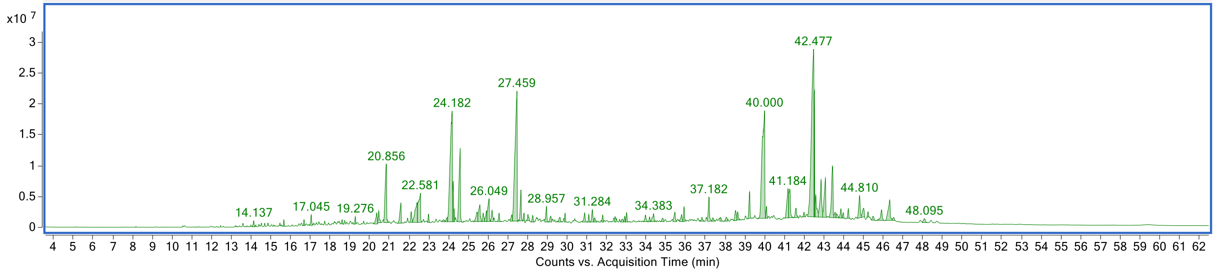

Figure S6. GC/MS chromatogram of the P7 sample extract

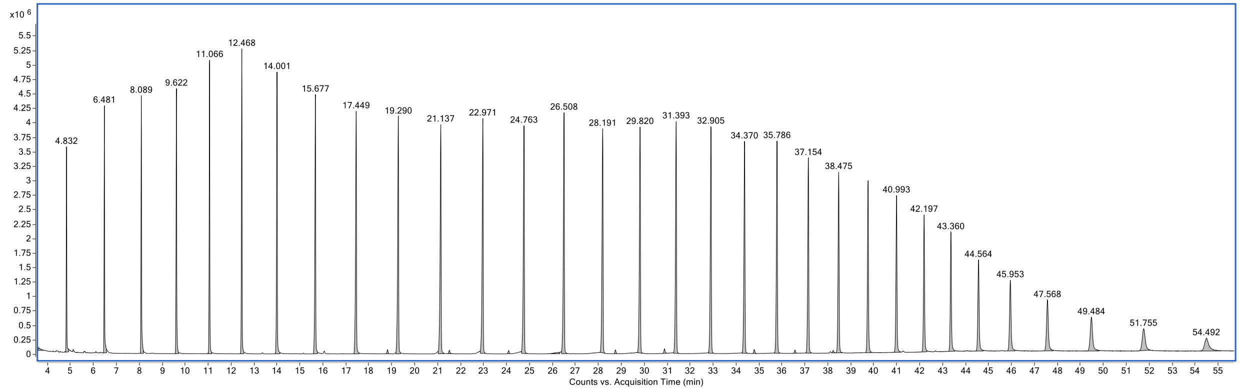

Figure S7. GC/MS chromatogram of the n-alkane mixture sample extract, C = 10 µg/ml. The chromatogram shows peaks for C9-C40 (the first two alkanes are missing due to solvent delay).

A. Examples of the identification algorithm applied to an identified compound

1. Identification of: 9-octadecen-1-ol, (Z)-

MassHunter: Score = 80 (substance initially identified) moving to the next stage of identification

AMDIS: To make it possible to use the retention indices, the C7-C40 mixture was injected and the retention times of alkanes were updated in the AMDIS program. The chromatogram of the cotton sample was analyzed according to the parameters given in the previous work [Dąbrowski, Ł. (2020). Evaluation of a Simplified Method for GC / MS Qualitative Analysis of Polycyclic Aromatic. *Molecules*, *25*, 3727]. The obtained extracted spectrum was sent to MS Search. If the results obtained from both programs were consistent with each other (and this was the case), the obtained RI (2064) was compared with those contained in the NIST17 database (2063±3) and the Mach Factor (955) was read. If there was no RI compliance (within the tolerance range of +/- 10), the compound would not be identified. The procedure would be repeated to find consistent identification results from the two programs and matching retention indices (from the chromatogram and the database).

2. Identification of octacosane

MassHunter: tetracosane ($C_{24}H_{50}$; Score: 75.32), heneicosane ($C_{21}H_{44}$; Score: 75.10), eicosane ($C_{20}H_{42}$; score: 77.71), etc. were identified.

AMDIS: octacosane only in the sixth position (MF = 891), so an analysis was performed using the PARADISE program (MF = 911) and after assessing RI compliance - this compound was selected as the identification result

Note: *Compounds with a MassHunter score below 75 were considered unidentified and were not subjected to further identification procedure. They could be treated as tentatively identified, but this would require redefining the established algorithm and necessarily confirming the results obtained.*

Sources of data collected in Tables S1-S8:

- Adverse effects – from PubChem [54] and Haz-Map [56] databases,
- LogP, WS, LogKoa, Kp – from the EpiSuite (ver.4.11, US EPA) program (experimental data and in the absence thereof, evaluated values),
- SE – PubChem and Haz-Map databases (experimental data), and in case of their absence, from the Pred-Skin 3.0 web application (LABMOL).

Table S1. Properties of identified compounds (alkanes) and the sample with the highest abundance (peak area). The colored rectangle (simplified heat map – SHM) corresponds to the order of magnitude of the peak area.

RT [min]	Compound name (synonym)	adverse effects	LogP	WS [mg/L]	LogKoa	Kp [cm/h]	SE	sample with the highest abundance (peak area) SHM
31.40	Pentacosane	SI, EI	12.62	0.0017	8.44	$4.0 \cdot 10^{3}$	no	F1
32.93	Hexacosane	SI	13.11	0.0017	9.17	$7.1 \cdot 10^{3}$	no	F5
34.39	Heptacosane	SI, EI	13.60	$2.8 \cdot 10^{-9}$	9.17	$1.2 \cdot 10^{4}$	no	F5
35.80	Octacosane	SI, EI	14.09	$8.8 \cdot 10^{-10}$	9.54	$2.2 \cdot 10^{4}$	no	F5
37.16	Nonacosane	SI, EI	14.58	$2.8 \cdot 10^{-10}$	9.91	$3.9 \cdot 10^{4}$	no	F8
38.48	Triacontane	SI, EI	15.07	$8.8 \cdot 10^{-11}$	10.27	$6.9 \cdot 10^{4}$	no	F3
39.72	Hentriacontane	SI, EI	15.57	$2.7 \cdot 10^{-11}$	10.60	$1.2 \cdot 10^{5}$	no	F1

RT – retention time, MW – molecular weight, VP – vapour pressure, LogP – octanol/water partition coefficient, LogKoa – octanol/air partition coefficient, WS – water solubility, EI – eye irritant, SI – skin irritant, I –irritant, SE – Skin sensitizer, DT – dermatotoxin, AQ – toxic to aquatic life, Edam – causes eye damage, ED – endocrine disruptor, Kp – dermal permeability coefficient; peak area: : $n \cdot 10^{5}$; : $n \cdot 10^{6}$; : $n \cdot 10^{7}$; : $n \cdot 10^{8}$.

Table S2. Properties of identified compounds (saturated fatty acids) and the sample with the highest abundance (peak area). The colored rectangle (simplified heat map – SHM) corresponds to the order of magnitude of the peak area.

RT [min]	Compound name (synonym)	adverse effects	LogP	WS [mg/L]	LogKoa	Kp [cm/h]	SE	sample with the highest abundance (peak area) SHM
10.66	nonanoic acid (pelargonic acid)	SI, EI, DT, AQ	3.42	284	7.60	0.038	no	P6
11.96	decanoic acid (capric acid)	SI, EI, AQ	4.09	61.8	8.35	0.0884	no	P3
15.11	dodecanoic acid (lauric acid)	SI, EI	4.6	8.41	8.02	0.134	no	F2
18.72	tetradecanoic acid (myristic acid)	SI, EI, AQ	6.11	0.183	9.91	0.942	yes	F2
22.37	hexadecanoic acid (palmitic acid)	SI, EI, AQ	7.17	0.04	10.26	3.32	no	F4
25.92	octadecanoic acid (stearic acid)	SI, EI	8.23	0.597	12.94	11.7	no	F4

RT – retention time, MW – molecular weight, VP – vapour pressure, LogP – octanol/water partition coefficient, LogKoa – octanol/air partition coefficient, WS – water solubility, EI – eye irritant, SI – skin irritant, I –irritant, SE – Skin sensitizer, DT – dermatotoxin, AQ – toxic to aquatic life, Edam – causes eye damage, ED – endocrine disruptor, Kp – dermal permeability coefficient; peak area: : $n \cdot 10^5$; : $n \cdot 10^6$; : $n \cdot 10^7$; : $n \cdot 10^8$.

Table S3. Properties of identified compounds (fatty alcohols) and the sample with the highest abundance (peak area). The colored rectangle (simplified heat map – SHM) corresponds to the order of magnitude of the peak area.

RT [min]	Compound name (synonym)	adverse effects	LogP	WS [mg/L]	LogKoa	Kp [cm/h]	SE	sample with the highest abundance (peak area) SHM
13.59	dodecan-1-ol	SI, EI, AQ	5.13	4.0	8.17	0.362	no	F9
17.03	tetradecan-1-ol	SI, EI, AQ	6.03	0.191	8.21	0.998	no	P7
20.83	hexadecane-1-ol	SI, EI, AQ	6.73	0.0411	9.90	2.04	no	P7
24.14	9-octadecen-1-ol, (Z)-	SI	7.50	0.02367	9.22	4.72	yes	P1
24.53	octadecan-1-ol (stearyl alcohol)	EI, AQ	7.72	0.0011	10.95	6.38	no	P7
40.14	octacosan-1-ol	SI, EI	12.63	$1.398\cdot10^{-7}$	13.07	$1.91\cdot10^{3}$	no	F8

RT – retention time, MW – molecular weight, VP – vapour pressure, LogP – octanol/water partition coefficient, LogKoa – octanol/air partition coefficient, WS – water solubility, EI – eye irritant, SI – skin irritant, I –irritant, SE – Skin sensitizer, DT – dermatotoxin, AQ – toxic to aquatic life, Edam – causes eye damage, ED – endocrine disruptor, Kp – dermal permeability coefficient; peak area: : $n\cdot10^{5}$; : $n\cdot10^{6}$; : $n\cdot10^{7}$; : $n\cdot10^{8}$.

Table S4. Properties of identified compounds (fatty acid esters) and the sample with the highest abundance (peak area). The colored rectangle (simplified heat map – SHM) corresponds to the order of magnitude of the peak area.

RT [min]	Compound name (synonym)	adverse effects	LogP	WS [mg/L]	LogKoa	Kp [cm/h]	SE	sample with the highest abundance (peak area) SHM
29.59	hexanedioic acid bis(2-ethylhexyl) ester (bis-2-ethylhexyl adipate)	SI, EI, AQ	8.12	0.78	12.87	3.23	no	P5
31.76	Hexadecanoic acid 2-hydroxy-1-(hydroxymethyl)ethyl ester (2-palmitoylglycerol)	I	5.63	0.1252	11.68	0.121	no	F8
42.96	Hexadecanoic acid hexadecyl ester (cetyl palmitate)	–	14.61	14.62	13.186	$1.62 \cdot 10^4$	no	F9
45.50	Hexadecanoic acid, octadecyl ester (lanolin)	–	15.60	$3.00 \cdot 10^{-11}$	13.932	$5.07 \cdot 10^4$	no	F9
48.83	Octadecanoic acid octadecyl ester (stearyl stearate)	–	16.58	$2.871 \cdot 10^{-12}$	14.663	$1.58 \cdot 10^5$	no	F9

RT – retention time, MW – molecular weight, VP – vapour pressure, LogP – octanol/water partition coefficient, LogKoa – octanol/air partition coefficient, WS – water solubility, EI – eye irritant, SI – skin irritant, I –irritant, SE – Skin sensitizer, DT – dermatotoxin, AQ – toxic to aquatic life, Edam – causes eye damage, ED – endocrine disruptor, Kp – dermal permeability coefficient; peak area: : $n \cdot 10^5$; : $n \cdot 10^6$; : $n \cdot 10^7$; : $n \cdot 10^8$.

Table S5. Properties of identified compounds (phthalate esters) and the sample with the highest abundance (peak area). The colored rectangle (simplified heat map – SHM) corresponds to the order of magnitude of the peak area.

RT [min]	Compound name (synonym)	adverse effects	LogP	WS [mg/L]	LogKoa	Kp [cm/h]	SE	sample with the highest abundance (peak area) SHM
20.36	diisobutyl phthalate	SI, EI, AQ, ED	4.11	6.2	8.14	0.0231	no	F1
22.11	dibuthyl phthalate	AQ, I - low	4.50	11.2	8.63	0.042	yes	F2

RT – retention time, MW – molecular weight, VP – vapour pressure, LogP – octanol/water partition coefficient, LogKoa – octanol/air partition coefficient, WS – water solubility, EI – eye irritant, SI – skin irritant, I –irritant, SE – Skin sensitizer, DT – dermatotoxin, AQ – toxic to aquatic life, Edam – causes eye damage, ED – endocrine disruptor, Kp – dermal permeability coefficient; peak area: : $n \cdot 10^5$; : $n \cdot 10^6$; : $n \cdot 10^7$; : $n \cdot 10^8$.

Table S6. Properties of identified compounds (fatty acid amides) and the sample with the highest abundance (peak area). The colored rectangle (simplified heat map – SHM) corresponds to the order of magnitude of the peak area.

RT [min]	Compound name (synonym)	adverse effects	LogP	WS [mg/L]	LogKoa	Kp [cm/h]	SE	sample with the highest abundance (peak area) SHM
26.09	Hexadecanamide (palmitamide)	AQ, I	5.71	0.2914	10.33	0.291	no	F6
29.28	9-octadecenamide, (Z-) (oleamide)	EI, SI, AQ	6.48	0.0459	10.97	0.836	yes	F1
29.66	Octadecanamide	SI, EI	6.70	0.0293	11.07	1.13	no	F6

RT – retention time, MW – molecular weight, VP – vapour pressure, LogP – octanol/water partition coefficient, LogKoa – octanol/air partition coefficient, WS – water solubility, EI – eye irritant, SI – skin irritant, I –irritant, SE – Skin sensitizer, DT – dermatotoxin, AQ – toxic to aquatic life, Edam – causes eye damage, ED – endocrine disruptor, Kp – dermal permeability coefficient; peak area: : $n \cdot 10^5$; : $n \cdot 10^6$; : $n \cdot 10^7$; : $n \cdot 10^8$.

Table S7. Properties of identified compounds (low molecular mass compounds) and the sample with the highest (peak area). The colored rectangle (simplified heat map – SHM) corresponds to the order of magnitude of the peak area.

RT [min]	Compound name (synonym)	adverse effects	LogP	WS [mg/L]	LogKoa	Kp [cm/h]	SE	sample with the highest abundance (peak area) SHM
7.05	Benzyl alcohol	EI, SI	1.1	$4.29\cdot10^{4}$	5.96	0.00209	yes	F5
9.80	2-propylheptan-1-ol	EI, SI, AQ, Edam	3.7	151.8	6.36	0.0596	no	F5
9.92	2-phenoxyethanol	EI, SI	1.16	$2.67\cdot10^{4}$	6.88	0.00155	yes	F2
10.10	Benzothiazole	EI, SI, DT, AQ	2.01	4300	6.826	0.00592	yes	F5

RT – retention time, MW – molecular weight, VP – vapour pressure, LogP – octanol/water partition coefficient, LogKoa – octanol/air partition coefficient, WS – water solubility, EI – eye irritant, SI – skin irritant, I –irritant, SE – Skin sensitizer, DT – dermatotoxin, AQ – toxic to aquatic life, Edam – causes eye damage, ED – endocrine disruptor, Kp – dermal permeability coefficient; peak area: : $n\cdot10^{5}$; : $n\cdot10^{6}$; : $n\cdot10^{7}$; : $n\cdot10^{8}$.

Table S8. Properties of identified compounds (other compounds) and the sample with the highest abundance (peak area). The colored rectangle (simplified heat map – SHM) corresponds to the order of magnitude of the peak area.

RT [min]	Compound name (synonym)	adverse effects	LogP	WS [mg/L]	LogKoa	Kp [cm/h]	SE	sample with the highest abundance (peak area) SHM
24.99	dimantine	SI, EI, AQ, DT	8.39	0.00888	9.124	12.6	no	F3
27.29	Tributyl acetylcitrate	–	4.29	5.0	12.101	0.00609	no	P7
31.99	Tetraethylene glycol monododecyl ether (Laureth-4)	SI, EI, AQ	6.37	12.3	13.508	0.00399	yes	P3
42.48	g-sitosterol	–	9.65	$4.632\cdot10^{-5}$	11.569	19	yes	F8
43.76	sigmast-4-en-3-one (sitostenone)	–	9.42	$2.284\cdot10^{-5}$	10.594	13.9	yes	F2

RT – retention time, MW – molecular weight, VP – vapour pressure, LogP – octanol/water partition coefficient, LogKoa – octanol/air partition coefficient, Kp - WS – water solubility, EI – eye irritant, SI – skin irritant, I –irritant, SE – Skin sensitizer, DT – dermatotoxin, AQ – toxic to aquatic life, Edam – causes eye damage, ED – endocrine disruptor, Kp – dermal permeability coefficient; peak area: : $n\cdot10^{5}$; : $n\cdot10^{6}$; : $n\cdot10^{7}$; : $n\cdot10^{8}$.
